# Lung cancer screening by nodule volume in Lung-RADS v1.1: negative baseline CT yields potential for increased screening interval

**DOI:** 10.1007/s00330-020-07275-w

**Published:** 2020-09-30

**Authors:** Mario Silva, Gianluca Milanese, Stefano Sestini, Federica Sabia, Colin Jacobs, Bram van Ginneken, Mathias Prokop, Cornelia M. Schaefer-Prokop, Alfonso Marchianò, Nicola Sverzellati, Ugo Pastorino

**Affiliations:** 1 Section of Radiology (Pad. Barbieri), Unit of Surgical Sciences, Department of Medicine and Surgery (DiMeC), University Hospital of Parma, University of Parma, Via Gramsci 14, 43126 Parma, Italy; 2 Department of Thoracic Surgery, IRCCS Istituto Nazionale dei Tumori, Milan, Italy; 3 Department of Radiology and Nuclear Medicine, Radboud University Medical Center, Nijmegen, The Netherlands; 4 Department of Radiology, Meander Medical Centre, Amersfoort, The Netherlands; 5 Department of Radiology, IRCCS Istituto Nazionale dei Tumori, Milan, Italy

**Keywords:** Lung neoplasms, Diagnostic screening programs, Solitary pulmonary nodule, Volume computed tomography, Lung volume measurements

## Abstract

**Objectives:**

The 2019 Lung CT Screening Reporting & Data System version 1.1 (Lung-RADS v1.1) introduced volumetric categories for nodule management. The aims of this study were to report the distribution of Lung-RADS v1.1 volumetric categories and to analyse lung cancer (LC) outcomes within 3 years for exploring personalized algorithm for lung cancer screening (LCS).

**Methods:**

Subjects from the Multicentric Italian Lung Detection (MILD) trial were retrospectively selected by National Lung Screening Trial (NLST) criteria. Baseline characteristics included selected pre-test metrics and nodule characterization according to the volume-based categories of Lung-RADS v1.1. Nodule volume was obtained by segmentation with dedicated semi-automatic software. Primary outcome was diagnosis of LC, tested by univariate and multivariable models. Secondary outcome was stage of LC. Increased interval algorithms were simulated for testing rate of delayed diagnosis (RDD) and reduction of low-dose computed tomography (LDCT) burden.

**Results:**

In 1248 NLST-eligible subjects, LC frequency was 1.2% at 1 year, 1.8% at 2 years and 2.6% at 3 years. Nodule volume in Lung-RADS v1.1 was a strong predictor of LC: positive LDCT showed an odds ratio (OR) of 75.60 at 1 year (*p* < 0.0001), and indeterminate LDCT showed an OR of 9.16 at 2 years (*p* = 0.0068) and an OR of 6.35 at 3 years (*p* = 0.0042). In the first 2 years after negative LDCT, 100% of resected LC was stage I. The simulations of low-frequency screening showed a RDD of 13.6–21.9% and a potential reduction of LDCT burden of 25.5–41%.

**Conclusions:**

Nodule volume by semi-automatic software allowed stratification of LC risk across Lung-RADS v1.1 categories. Personalized screening algorithm by increased interval seems feasible in 80% of NLST eligible.

**Key Points:**

*• Using semi-automatic segmentation of nodule volume, Lung-RADS v1.1 selected 10.8% of subjects with positive CT and 96.87 relative risk of lung cancer at 1 year, compared to negative CT.*

*• Negative low-dose CT by Lung-RADS v1.1 was found in 80.6% of NLST eligible and yielded 40 times lower relative risk of lung cancer at 2 years, compared to positive low-dose CT; annual screening could be preference sensitive in this group.*

*• Semi-automatic segmentation of nodule volume and increased screening interval by volumetric Lung-RADS v1.1 could retrospectively suggest a 25.5–41% reduction of LDCT burden, at the cost of 13.6–21.9% rate of delayed diagnosis.*

**Electronic supplementary material:**

The online version of this article (10.1007/s00330-020-07275-w) contains supplementary material, which is available to authorized users.

## Introduction

Lung cancer screening (LCS) by low-dose computed tomography (LDCT) allows for early diagnosis of lung cancer (LC), resulting in a significant reduction of LC mortality [1–4]. Most trials, including the National Lung Screening Trial (NLST), applied annual screening frequency, which was derived from modelling analysis [5, 6].

The efficiency of annual screening depends on an individual risk of LC, which varies substantially even among subjects with risk factors. In one analysis of NLST population, Kovalchik et al showed that the number needed to screen increased by 60-fold for the lowest risk quintile compared with that for the highest risk quintile [7]. The efficiency of annual screening scans was substantially reduced in the lowest-risk subgroup. To improve cost-effectiveness, a Canadian cost-effectiveness modelling study [8] and post hoc analysis of NLST data [9, 10] suggested to increase screening interval after negative LDCT. Two positive European trials showed reduction of LC mortality by protocols that used longer screening intervals and nodule volumetry, but had populations with lower risk than NLST [2, 3].

To date, studies addressing LC risk by LDCT result in NLST eligible are based on linear measurement of nodule diameter [6]. Nodule characteristics were the strongest predictors of LC in several retrospective analyses in NLST population [9, 10], thus representing an option for systematic improvement of screening efficiency. Risk stratification might vary between linear measurements and nodule volumetry, especially when different definitions of diameters are applied (e.g. mean diameter or maximum diameter) [11]. Nodule volumetry is deemed the most accurate predictor of LC risk [12], and it is proposed by updated quality assurance initiatives and guidelines, for both optimal baseline classification and longitudinal characterization of growth (e.g. volume doubling time) [13–19].

The linear algorithm of the American College of Radiology (ACR) Lung CT Screening Reporting & Data System (Lung-RADS) [20] was updated in 2019 to include conversion of diameter into volume of a sphere (version 1.1) [21]. The distribution and LC risk of Lung-RADS v1.1 categories by semi-automatic nodule volume is not published in the peer review literature. Also, it is unknown whether these categories might allow for post-test risk stratification for referral to longer intervals between screening rounds.

The aims of this study were to report the distribution of Lung-RADS v1.1 volumetric categories in a population of NLST-eligible subjects and to analyse LC outcomes within 3 years for exploring personalized algorithm for LCS.

## Materials and methods

The Multicentric Italian Lung Detection (MILD) study was a prospective randomized controlled LCS trial launched in 2005 (ethics committee approval INT 53/05; ClinicalTrials.gov NCT02837809). Informed consent was obtained from eligible subjects before participation in the MILD study. The sponsors had no role in conducting and interpreting the study [22].

MILD eligibility criteria were as follows: age > 49 years, smoking history ≥ 20 pack-years, current or former smoker with smoking cessation within 10 years prior to study enrolment and no oncologic history in 5 years before recruitment. The overall population of MILD study included 4099 participants, with 2376 subjects in the intervention arm (median age 58 years, median 39 pack-years, men 68%) [23]. MILD study population showed a relatively lower-risk profile than the NLST population, as a consequence of lower threshold in age and smoking eligibility criteria [1]. In this study, we extracted a higher-risk population from the MILD study by applying the NLST eligibility criteria to the intervention arm: age ≥ 55 years and pack-years ≥ 30 [1]. Fifty-three percent (1248/2376) of MILD subjects were NLST eligible.

Selected pre-test metrics were gender, smoking status, percentage of predicted forced expiratory volume in 1 s (FEV1%_pred_, with threshold at 0.9) and the ratio between forced expiratory volume in 1 s and forced vital capacity (FEV1/FVC, with threshold at 0.7) [24].

Baseline LDCT result was defined as follows.

### Baseline LDCT result

Baseline LDCT results of the 1248 NLST-eligible subjects were retrospectively analysed by an advanced workstation for LCS (CIRRUS Lung Screening), featuring computer-aided detection (CAD) and semi-automated segmentation for direct measurement of nodule volume, for both solid and subsolid nodules. All CAD marks were jointly reviewed by two experienced thoracic radiologists (8 years and 11 years of experience in LC screening, each with > 10.000 LDCTs) [25]. The segmentation of nodule volume was checked and adjusted, if necessary, using semi-automatic parameter tuning (density threshold and irregularity metrics; manual contouring not allowed). The segmented total nodule volume and volume of the solid component of a part-solid nodule were used for the present study.

Volumetric thresholds were applied at baseline LDCT according to Lung-RADS v1.1, and management suggestions were used to establish three major categories of baseline LDCT result:

- *Negative (categories 1 and 2: annual screening suggested)*: no nodule, solid or part-solid nodule with whole volume < 113 mm^3^ (diameter 6 mm), or non-solid nodule < 14,137 mm^3^ (diameter 30 mm)
- *Indeterminate (category 3: 6-month LDCT suggested)*: solid nodule 113–268 mm^3^ (diameter 6–8 mm), part-solid nodule with whole volume ≥ 113 mm^3^ and solid component < 113 mm^3^, or non-solid nodule ≥ 14,137 mm^3^ (diameter 30 mm)
- *Positive (categories 4A and 4B: early recall or work-up)*: solid nodule > 268 mm^3^ or part-solid nodule with solid component ≥ 113 mm^3^

### Statistical analyses

The distribution of each variable was described in the whole population and, according to the diagnosis of LC, at each pre-defined time interval. Frequencies were reported as percentage.

The primary outcome was the number of LC diagnosed within 1 year, 2 years and 3 years after baseline. The secondary outcome was the stage of LC. The outcomes were collected during the MILD trial, which included systematic prospective registration of LC stage, type of resection and histology.

In this analysis, the cumulative frequency of LC was calculated at the three pre-defined time intervals. The cumulative frequency of LC was tested against pre-test metrics or baseline LDCT result. The relative risk (RR) was calculated by the ratio between the risks of LC in each LDCT group for each parameter, at each time interval. The RR of pre-test parameters was calculated using the category at lower risk as reference. The RR of indeterminate and positive LDCT results was calculated using negative LDCT result as reference.

Kaplan-Meier curves were used to test the probability of LC for each time interval by unadjusted hazard ratio (HR). The odds ratio (OR) by univariate and multivariable models was calculated at each time interval.

The reduction of LDCT burden was calculated by simulation of 4 screening algorithms for increased round interval by post-test risk stratification with volumetric nodule categories of Lung-RADS v1.1 (Table 1). The simulated algorithms stemmed from the annual algorithm of Lung-RADS v1.1 and were developed to encompass the following:

- *Biennial* or *triennial* frequency for negative LDCT
- *Six-month recall* or *annual recall* for indeterminate LDCT

**Table 1:** Flow chart detailing the 4 simulations of screening algorithms with increased round interval

	Negative baseline LDCT	Indeterminate baseline LDCT	Positive baseline LDCT
Algorithm 1	LDCT in 2 years	LDCT in 6 months	LDCT in ≤ 3 months
Algorithm 2	LDCT in 2 years	LDCT in 1 year	LDCT in ≤ 3 months
Algorithm 3	LDCT in 3 years	LDCT in 6 months	LDCT in ≤ 3 months
Algorithm 4	LDCT in 3 years	LDCT in 1 year	LDCT in ≤ 3 months

Positive LDCT was always intended for *short-term recall* within 3 months.

An ideal 100% adherence was assumed throughout the LCS period, and the recall rate at incidence rounds was calculated according to the literature (range 3–14%) [26]. Increasing screening interval is expected to result in some degree of delayed LC diagnosis; therefore, the rate of delayed diagnosis (RDD) was calculated as follows: ratio between the number of LC diagnosed within the proposed interval (6 months or 1 year for indeterminate, 2 years or 3 years for negative) and the total number of LC in the full cycle of screening (2 years or 3 years). The proportion of LC in stage I was calculated for each algorithm at each time interval.

Statistical analysis was performed by Statistical Analysis System software (version 9.4; SAS Institute) and MedCalc statistical software (version 17.4; MedCalc Software Bvba).

## Results

**Fig. 1:**
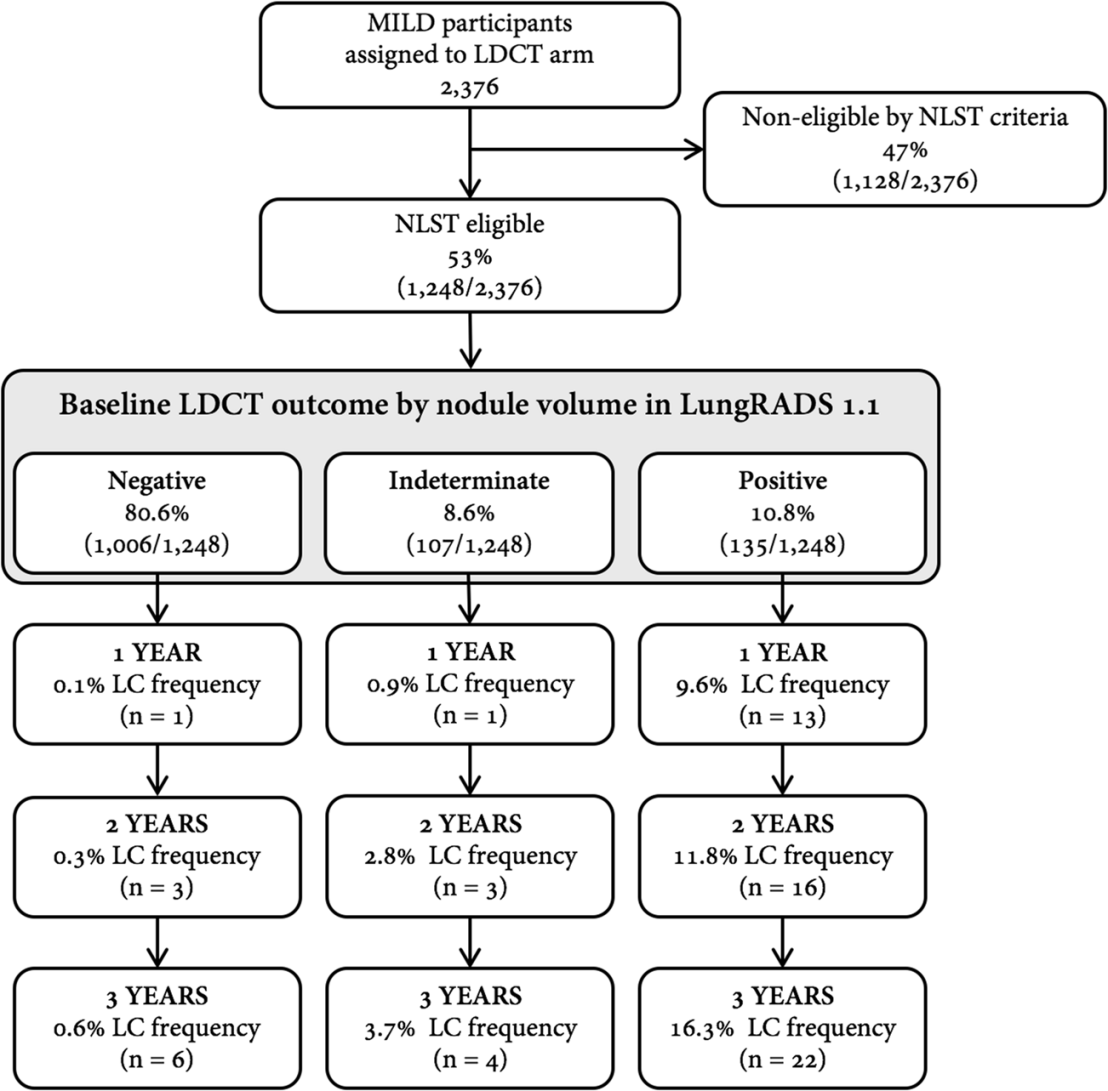
The flow diagram details the selection of high-risk subjects, classification by nodule volume in ACR Lung-RADS v1.1 at baseline low-dose computed tomography and relevant cumulative distribution of lung cancer

In the 1248 NLST-eligible subjects (Fig. 1), cumulative LC frequency was 1.2% at year 1, 1.8% at year 2 and 2.6% at year 3. Negative LDCT at baseline was seen in 1006 subjects (80.6%; 715 without nodule, 175 with solid nodule and 116 with non-solid nodule), indeterminate in 107 subjects (8.6%; 101 with solid nodules and 6 with part-solid nodules) and positive in 135 subjects (10.8%; 111 with solid nodules and 24 with part-solid nodules).

### Diagnosis of LC

The probability of LC at years 1, 2 and 3 was higher in subjects with either positive or indeterminate baseline LDCT (Table 2).

**Table 2:** Frequency of LC and odds ratio (OR) at 1 year, 2 years and 3 years, according to the pre-test metrics and baseline LDCT result

	Total	1 year				2 years				3 years			
		No LC	LC	RR	OR [95% CI] (*p*)	No LC	LC	RR	OR [95% CI] (*p*)	No LC	LC	RR	OR [95% CI] (*p*)
	1248 (100%)	1233 (98.8%)	15 (1.2%)			1226 (98.2%)	22 (1.8%)			1216 (97.4%)	32 (2.6%)		
Pre-test metrics													
Gender													
Female*	349 (28)	349 (100)	0 (0)			347 (99.4)	2 (0.6)			345 (98.9)	4 (1.1)		
Male	899 (72)	884 (98.3)	15 (1.7)	N.A.	N.A.	879 (97.8)	20 (2.2)	3.88	3.92 [0.92–16.67] (0.0654)	871 (96.9)	28 (3.1)	2.72	2.77 [0.97–7.96] (0.0582)
Smoking status													
Former*	421 (33.7)	417 (99)	4 (1)			414 (98.3)	7 (1.7)			412 (97.9)	9 (2.1)		
Current	827 (66.3)	816 (98.7)	11 (1.3)	1.40	1.41 [0.45–4.44] (0.5624)	812 (98.2)	15 (1.8)	1.09	1.10 [0.45–2.67] (0.8426)	804 (97.2)	23 (2.8)	1.30	1.31 [0.6–2.86] (0.498)
FEV1%_pred_													
> 90%*	809 (65.1)	806 (99.6)	3 (0.4)			803 (99.3)	6 (0.7)			799 (98.8)	10 (1.2)		
≤ 90%	435 (34.9)	423 (97.2)	12 (2.8)	7.44	7.62 [2.14–27.16] (0.0017)	419 (96.3)	16 (3.7)	4.96	5.04 [1.98–12.81] (0.0007)	413 (94.9)	22 (5.1)	4.09	4.26 [2–9.07] (0.0002)
Missing	4	4	0			4	0			4	0		
			–				–				–		
FEV1/FVC													
> 70%*	883 (71.4)	878 (99.4)	5 (0.6)			875 (99.1)	8 (0.9)			872 (98.8)	11 (1.2)		
≤ 70%	354 (28.6)	345 (97.5)	9 (2.5)	4.49	4.58 [1.52–13.77] (0.0067)	342 (96.3)	13 (3.7)	4.05	5.04 [1.98–12.81] (0.0016)	336 (94.9)	18 (5.1)	4.08	4.25 [1.99–9.09] (0.0002)
Missing	11	10	1			10	1			8	3		
			–				–				–		
Baseline LDCT outcome													
Negative*	1006 (80.6)	1005 (99.9)	1 (0.1)			1003 (99.7)	3 (0.3)			1000 (99.4)	6 (0.6)		
Indeterminate	107 (8.6)	106 (99.1)	1 (0.9)	9.40	9.42 [0.60–148.44] (0.1128)	104 (97.2)	3 (2.8)	9.40	9.47 [1.93–46.53] (0.0059)	103 (96.3)	4 (3.7)	6.27	6.36 [1.81–22.39] (0.0042)
Positive	135 (10.8)	122 (90.4)	13 (9.6)	96.87	102.06 [13.49–772.19] (< 0.0001)	119 (88.2)	16 (11.8)	39.74	42.58 [12.48–145.23] (< 0.0001)	113 (83.7)	22 (16.3)	27.32	29.98[12.21–73.62] (< 0.0001)

*Reference (ref) category for calculation of relative risk (RR) and odds ratio (OR) with 95% of confidence interval (95% CI)

**Fig. 2:**
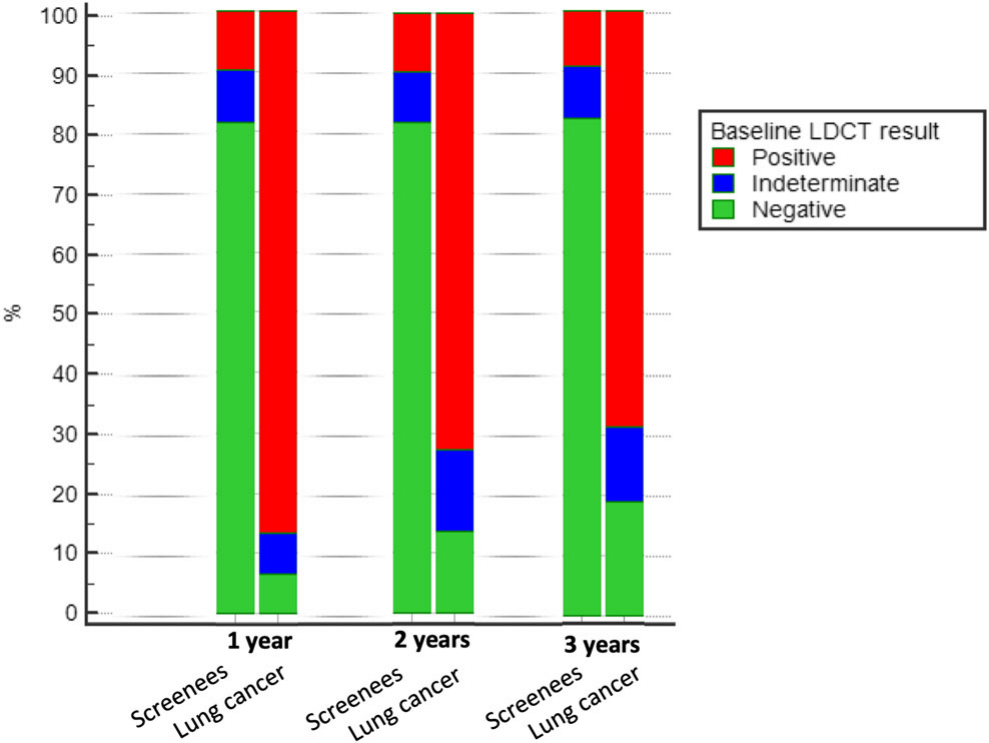
Relative distribution of LDCT result and LC diagnosis at 1 year, 2 years and 3 years

At year 1, 9.6% (13/135) frequency of LC was seen for positive LDCT, 0.9% (1/107) for indeterminate and 0.1% (1/1006) for negative; 86.6% (13/15) of LC at year 1 was seen after a positive LDCT (Fig. 2). The frequency of LC after negative baseline LDCT remained as low as 0.3% at 2 years and 0.6% at 3 years.

**Fig. 3:**
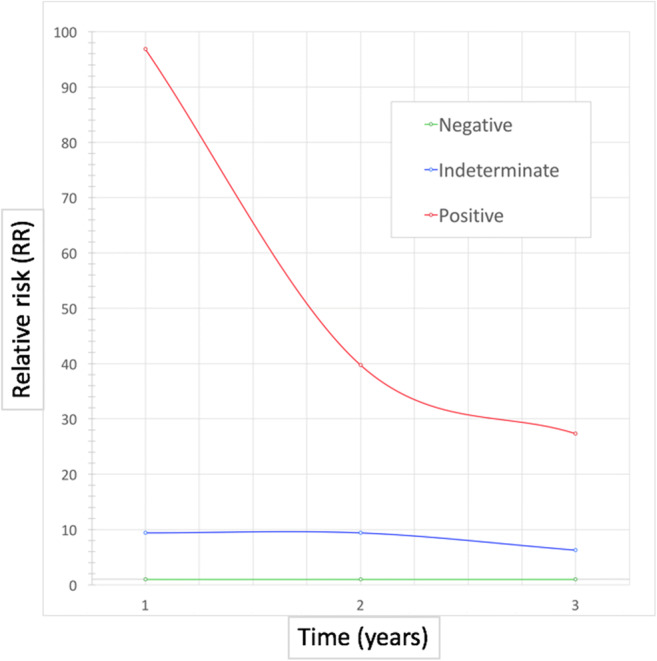
Time-resolved relative risk (RR) for lung cancer, according to the baseline LDCT result

**Fig. 4:**
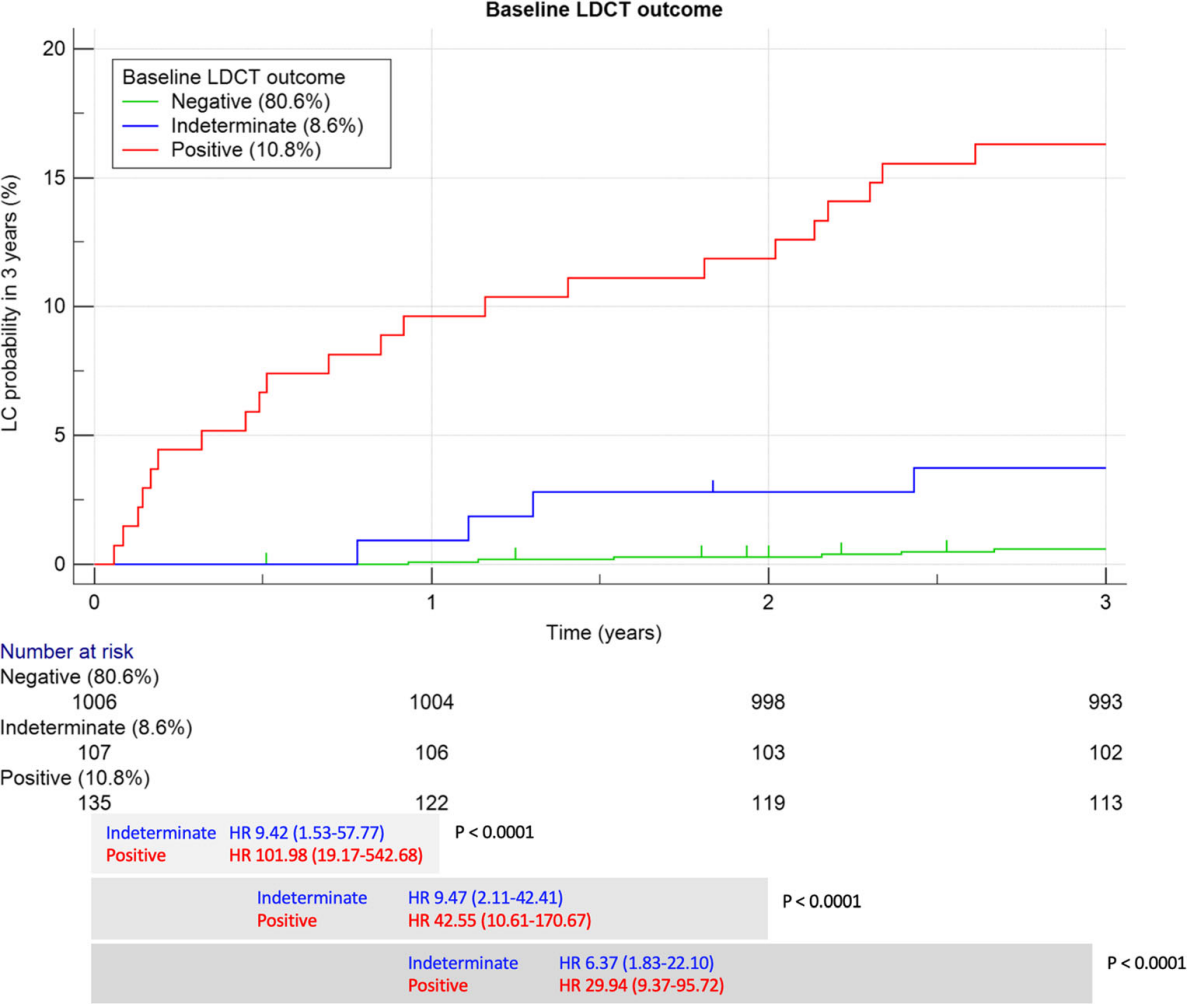
Kaplan-Meier curve for diagnosis of LC through 3 years, according to the baseline LDCT result. The bottom section shows the HR by each pre-defined time point analysis of LC probability

LDCT result was the strongest predictor of LC through 3 years (Table 2): positive baseline LDCT yielded a RR of 96.87 at year 1, with a progressive decrease through year 2 (RR 39.74) and year 3 (RR 27.32) (Fig. 3). Indeterminate LDCT was associated with a RR of 9.40 at years 1 and 2, slightly decreasing to 6.27 at year 3. The HR for LC was highest in year 1 after positive LDCT and decreased through years 2 and 3 (Fig. 4).

#### Univariate analysis of LC risk

Baseline LDCT result was a predictor of LC risk, with greater magnitude than any pre-test metric (Table 2): analysis at 1-year interval showed an OR of 102.06 (95% CI 13.49 to 772.19; *p* < 0.0001) decreasing in analyses at 2-year and 3-year intervals. Indeterminate LDCT steadily yielded an OR above 9 at 1-year and 2-year intervals, while decreasing to 6.36 at 3-year interval.

##### Multivariable analysis of LC risk

In multivariable analysis, baseline LDCT result remained a predictor of LC risk (Table 3). Positive LDCT showed an OR of 75.60 (95% CI 9.86 to 579.83; *p* < 0.001) for analysis at 1-year interval and decreased at 2-year and 3-year intervals. Indeterminate LDCT yielded a significant OR of 9.16 (95% CI 1.86 to 45.13; *p* = 0.0068) through a 2-year interval and maintained an OR of 6.35 (95% CI 1.80 to 22.42; *p* = 0.0042) at 3-year interval.

**Table 3:** Multivariate analysis of risk of LC diagnosis at 1 year, 2 years and 3 years, according to the pre-test metrics and baseline LDCT result

	1 year		2 years		3 years	
	OR (95% CI)	*p*	OR (95% CI)	*p*	OR (95% CI)	*p*
Pre-test metrics						
Gender*						
Female	ref		ref		ref	
Male	N.A.	–	2.89 (0.67–12.38)	0.1560	2.04 (0.71–5.88)	0.1629
Smoking status						
Former	ref		ref		ref	
Current	1.55 (0.49–4.93)	0.4623	1.19 (0.48–2.95)	0.7155	1.56 (0.69–3.53)	0.2884
FEV1%_pred_						
> 90%	ref		ref		ref	
≤ 90%	3.29 (0.82–13.23)	0.0958	2.37 (0.84–6.68)	0.1039	1.77 (0.76–4.10)	0.1866
FEV1/FVC						
> 70%	ref		ref		ref	
≤ 70%	2.06 (0.62–6.81)	0.2407	2.27 (0.86–5.99)	0.0982	2.74 (1.20–6.23)	0.0042
Baseline LDCT result						
Negative	ref		Ref		ref	
Indeterminate	7.90 (0.50–125.03)	0.1445	9.16 (1.86–45.13)	0.0068	6.35 (1.80–22.42)	0.0042
Positive	75.60 (9.86–579.83)	< 0.0001	31.01 (8.93–107.62)	< 0.0001	21.36 (8.49–53.79)	< 0.0001

*OR* odds ratio, *CI* confidence interval

*OR could not be calculated because of the absence of LC in one category of the selected metric

A selective analysis of risk by pre-test metrics was performed in the group with negative LDCT: no outstanding pre-test variable was found for risk stratification (all *p* > 0.05, at any time point).

### Stage of LC

Stage and histology of LC are detailed by year and LDCT category in Table 4. After a positive LDCT, 13 LC subjects were diagnosed in the first year with 30.8% of stage I LC (4 stage I, 2 stage II, 3 stage III, 4 stage IV) and further 9 were diagnosed in the second and third years with 55.6% stage I LC (5 stage I and 4 stage IV). Otherwise, early-stage LC was most common after negative baseline LDCT (83% stage I and 17% stage IV) or indeterminate LDCT (75% stage I and 25% stage II), through the 3 years of this analysis.

**Table 4:** Distribution of the stage and histology of LC through the 3-year interval, according to the baseline LDCT result

	Year 1				Year 2				Year 3				Year 1				Year 2				Year 3			
	Stage												Histology											
	I	II	III	IV	I	II	III	IV	I	II	III	IV	ADC	SCC	SCLC	Other	ADC	SCC	SCLC	Other	ADC	SCC	SCLC	Other
Negative	1	–	–	–	2	–	–	–	2	–	–	1	1	–	–	–	1	1	–	–	2	1	–	–
Indeterminate	–	1	–	–	2	–	–	–	1	–	–	–	1	–	–	–	2	–	–	–	1	–	–	–
Positive	4	2	3	4	2	–	–	1	3	–	–	3	5	5	–	3	2	1	–	–	4	–	–	2

### Personalized LCS algorithm

The overall RDD ranged from 13.6 to 21.9%, with a rate of stage I between 75 and 100% among the potentially delayed LC diagnoses (Table 5). Algorithm 1 (2-year interval for negative LDCT, 6-month follow-up for indeterminate LDCT) showed the lowest RDD (13.6%) with the potential 25.5–26.5% reduction of LDCT over 2 years, compared to annual benchmark. Algorithm 4 (3-year interval for negative LDCT, 1-year follow-up for indeterminate LDCT) showed not only the highest potential reduction of LDCT burden (40.6–41%) but also the highest RDD (21.9%) over 3 years, compared to annual benchmark.

**Table 5:** Simulation of lung cancer screening management by nodule volume in ACR Lung-RADS v1.1 with the annual screening interval and four proposed increased screening interval algorithms

	Negative LDCT	Indeterminate LDCT	Positive LDCT	Overall	Reduction of LDCT burden compared with annual screening		Sensitivity (%)	Specificity (%)	Positive predictive value (%)	Negative predictive value (%)
	1006	107	135		3% recall	14% recall				
ACR Lung-RADS v1.1 (2-year reference)^a^	Annual	6 months	Short term		Reference, 4061	Reference, 4335	94.1	90.3	11.9	99.9
Delayed diagnosis (proportion of stage I)	1 (100%)	0	0	1 (100%)						
RDD	4.5% (1/22)	–	–	4.5% (1/22)						
Algorithm 1^a^	Biennial	6 months	Short term		25.5% (3025/4061)	26.5% (3189/4335)	84.2	90.3	11.9	99.7
Delayed diagnosis (proportion of stage I)	3 (100%)	0	0	3 (100%)						
RDD	13.6% (3/22)	–	–	13.6% (3/22)						
Algorithm 2^a^	Biennial	Annual	Short term		28.2% (2918/4061)	28.9% (3082/4335)	80.0	90.3	11.9	99.6
Delayed diagnosis (proportion of stage I)	3 (100%)	1 (0%)	0	4 (75%)						
RDD	13.6% (3/22)	4.5% (1/22)	–	18.2% (4/22)						
ACR Lung-RADS v1.1 (3-year reference)^b^	Annual	6-month recall	Short term		5346	5758	95.7	90.8	16.3	99.9
Delayed diagnosis (proportion of stage I)	1 (100%)	0	0	1 (100%)						
RDD	3.1% (1/32)	–	–	3.1% (1/32)						
Algorithm 3^b^	Triennial	6-month recall	Short term		38.6% (3174/5346)	39.2% (3503/5758)	78.6	90.7	16.3	99.5
Delayed diagnosis (proportion of stage I)	6 (83.3%)	0 (0%)	0	5 (100%)						
RDD	18.8% (6/32)	–	–	18.8% (6/32)						
Algorithm 4^b^	Triennial	Annual	Short term		40.6% (3174/5346)	41.0% (3396/5758)	75.9	90.7	16.3	99.4
Delayed diagnosis (proportion of stage I)	6 (83.3%)	1 (0%)	0	6 (83.3%)						
RDD	18.8% (6/32)	3% (1/32)	–	21.9% (7/32)						

ACR Lung-RADS v1.1 with annual screening interval serves as reference for simulation of LDCT reduction by increased screening interval algorithms. Negative and indeterminate LDCT results were gathered for calculation of sensitivity, specificity, positive predictive value and negative predictive value

^a^Twenty-two LC subjects diagnosed in 2 years; estimated 4102 LDCT screening by annual algorithm in 2 years

^b^Thirty-two LC subjects diagnosed in 3 years; estimated 5350 LDCT screening by annual algorithm in 3 years

## Discussion

Nodule volume by semi-automatic software allowed stratification of LC risk across Lung-RADS v1.1 categories. This method classified 80.6% negative LDCT with 0.1% frequency of LC at 1 year, notably 10 times lower than the mean risk of LC in NLST eligible. Multivariable analysis showed that nodule volume outstands as a risk factor above pre-test metrics, especially for stratification of 1-year risk of LC. The proposed algorithms for increased interval screening showed a potential 25.5–41% reduction of LDCT burden, at the risk of 13.6–21.9% RDD.

To the best of our knowledge, this is the first analysis of Lung-RADS v1.1 using semi-automatic volume measurement.

### Post-test risk by Lung-RADS v1.1

The population of this study was retrospectively selected by NLST criteria, and indeed, the frequency of LC was comparable to original NLST results at year 1 (1.2% here vs 1.0% NLST), year 2 (1.8% vs 1.8%) and year 3 (2.6% vs 2.7%) [1].

The proportion of nodule categories and their LC risk estimated in the latest Lung-RADS v1.1 are different from the values measured in this study. The Lung-RADS v1.1 estimated 90% frequency of categories 1 and 2 with < 1% probability of LC in 1 year [21]. We measured the 80.6% frequency for categories 1 and 2, with as low as 0.1% risk of LC at year 1. Furthermore, we included also measurement of risk at year 2 (0.3%) and year 3 (0.6%). Caverly et al [27] reported that LC risk ≤ 0.3% would make LCS a preference-sensitive practice. This practice was endorsed by Robbins et al [28] in a retrospective analysis of NSLT data by diameter classification. Our study displays that a 0.3% risk is found 2 years after negative LDCT, suggesting that biennial screening might be proposed after negative baseline LDCT by Lung-RADS v1.1. This personalized approach might be assisted by counselling for decision aid and continuous individualized risk assessment (pre-test and post-test risks) [29, 30].

The Lung-RADS v1.1 estimated a 5% frequency of category 3, with 1–2% probability of LC. We measured the 8.6% frequency of category 3 with a 0.9% risk of LC in year 1. A single cancer in this category was diagnosed at 10 months, which is consistent with the 6-month recall proposed by Lung-RADS v1.1. The 6-month recall should be preferred over shorter follow-up (e.g. 3 months) [31] because the relatively small volume of this category is prone to low accuracy in short-term volumetry for assessment of actual growth [32].

The Lung-RADS v1.1 estimated the 4% frequency of category 4, whereas we measured the 10.8% frequency of this category. Such discrepancy might be accountable to the direct measurement of nodule volume by semi-automatic software as opposed to the theoretical conversion of diameter into geometrical sphere. We found a 9.6% risk of LC in category 4 compared with the expected > 5% reported in Lung-RADS v1.1. Baseline nodule volume by semi-automatic segmentation allowed remarkable post-test risk stratification in year 1, with a RR of 96.87 for category 4 compared with category 1 or 2.

The negative predictive value of Lung-RADS v1.1 in our selection was comparable to the 2-year performance of the NELSON volumetric protocol [33]. However, the 268 mm^3^ positive threshold of Lung-RADS v1.1 likely predicated a lower positive predictive value compared with that of the NELSON protocol (500 mm^3^ threshold at baseline and subsequent characterization by volume doubling time) [34, 35]. The geometrical volumetric threshold of Lung-RADS v1.1 could be further developed by comparison with NELSON reference, which is based on the direct measurement of nodule volume by semi-automatic software and is currently validated in over 10 years of follow-up [36]. It is anticipated that future studies will test a blend of Lung-RADS v1.1 category and volume doubling time to improve its accuracy.

### Simulation of increased screening interval

The standard of reference from NLST showed that efficiency of annual screening is variable even among subjects at high risk [7], for whom increased screening interval might be hypothesized in selected cases. Post hoc analysis of the NLST dataset brought a wealth of data for optimization of LCS algorithm [37], notably increased diameter thresholds [37] (featured in Lung-RADS v1.0) and evidence of safe feasibility of biennial screening [9, 10]. Patz et al [9] retrospectively modelled biennial screening with a 0.48% frequency of LC in 2 years after negative baseline LDCT by linear measurement (< 4 mm). The same authors also reported a 1.1% frequency of LC in 3 years after negative baseline LDCT. Our results show that a semi-automatic volume in category 1 or 2 of Lung-RADS v1.1 (e.g. negative LDCT result) selects even a lower frequency of LC (0.3% at 2 years and 0.6% at 3 years). These figures came along with the 80.6% of population with negative baseline LDCT, which points to a potentially remarkable reduction of LDCT burden by longer-than-annual screening interval. With this purpose, we simulated several scenarios of increased screening interval and compared these with the reference standard of annual screening by Lung-RADS v1.1.

The proposed algorithms showed a reduction of LDCT burden ranging from 25 to 41% through years 2 and 3, reflecting a potential-paralleled reduction in both costs and cumulative radiation burden. Our biennial simulation is aligned with the prospective results from the interval randomization of MILD, which showed an overall 38% reduction of LDCT burden in the biennial arm, without detrimental effect [38]. The biennial strategy might also help with the growing demand of capacity for steadily increasing utilization of LCS [39]. Biennial follow-up is endorsed by the National Health Service (NHS) England in their protocol for implementation of targeted lung health check programme at population level [40]. The NHS England proposed a reference volume < 80 mm^3^ for biennial follow-up of solid nodules, which is smaller than the < 113 mm^3^ reference analysed in our paper. Noteworthy, the performance of either threshold might vary significantly depending on the software for semi-automatic segmentation of volume [41].

Otherwise, the triennial simulations appeared quite hazardous because they were associated with up to 21.9% delayed diagnosis, with potential overlooking of a substantial proportion of stage I LC. Despite this figure is quite close to the frequency of overdiagnosis modelled on the NLST data [42], however, we cannot assume such overlapping. A NELSON report showed that a 2.5-year interval is associated with a significant increase of interval cancer and substantial stage shift [43]. We confirm that LDCT result alone does not allow safe selection for triennial screening interval. Complementary approach to LCS by integrating biological risk stratification might allow safe application of biennial or triennial screening interval [44]. Algorithm 4 of this paper was prospectively tested in the bioMILD trial (NCT02247453) where triennial LDCT was proposed along with personalized risk stratification by microRNA signature classifier (MSC). The bioMILD trial started in 2013 (4119 subjects, 50–75 years, ≥ 30 pack-years), with a semi-automatic segmentation of nodule and volumetric thresholds closely overlapping the most recent Lung-RADS v1.1. The preliminary results of bioMILD trial reported that the personalized interval of LCS every 3 years is safe when both semi-automatic nodule volume and MSC render negative result at baseline [45].

This study has some limitations. We grouped Lung-RADS category 4 under the definition of positive LDCT result and did not detail the granularity of categories 4A, 4B and 4X. We did not assess nodule-specific cancer rate but measured subject-specific cancer rate; this approach was intended to better serve in screening practice. The small population might have hindered a significant association of pre-test metrics with a risk of LC; nonetheless, it could show robust post-test risk stratification by Lung-RADS v1.1. The retrospective nature of this study hampers the actual estimation of stage shift and interval cancers [43]. Nonetheless, such a retrospective approach provided a picture of the nodule distribution under cutting-edge technological conditions for semi-automatic nodule volume and recently updated Lung-RADS v1.1. The present study did not account for further imaging findings (e.g. emphysema, interstitial lung abnormalities, pleural plaques), which might confer an additional post-test risk beyond nodule [40, 46–49]. Finally, the proposed algorithms might result in different outcomes when applied out of the present population and methods (e.g. volume LDCT analysed by CAD); therefore, caution is granted before application of these findings. The number of LC is quite low in groups with negative or intermediate LDCT, and this figure should be carefully interpreted because different absolute values might be observed in other settings. Prospective trials are needed to test whether the trade-off of longer screening intervals after negative LDCT will lead to a reduction in the number of lung cancer deaths avoided [50, 51].

In conclusion, this study represents the first analysis of Lung-RADS v1.1 category distribution using semi-automatic software for segmentation of nodule volume on thin-slice LDCT. This volumetric approach led to personalized stratification of LC risk among NLST-eligible subjects, and notably, the risk of LC in Lung-RADS v1.1 category 1 or 2 was substantially lower than that in category 3. Lung-RADS v1.1 categories showed that potential for longer than 1-year screening interval in up to 80% of NLST-eligible subjects.

## Electronic supplementary material

ESM 1 (DOCX 1297 kb)

## Abbreviations

ACR: American College of Radiology
BTS: British Thoracic Society
CAD: Computer-aided detection
HR: Hazard ratio
LC: Lung cancer
LCS: Lung cancer screening
LDCT: Low-dose computed tomography
Lung-RADS v1.1: Lung CT Screening Reporting & Data System version 1.1
MILD: Multicentric Italian Lung Detection
MSC: MicroRNA signature classifier
NHS: National Health Service
NLST: National Lung Screening Trial
OR: Odds ratio
RDD: Rate of delayed diagnosis
RR: Relative risk
