# Supplementary material for: Lung cancer screening by nodule volume in Lung-RADS v1.1: negative baseline CT yields potential for increased screening interval
Source: Eur Radiol. 2020 Sep 30;31(4):1956–68. doi: 10.1007/s00330-020-07275-w (PMC7979670; doi:10.1007/s00330-020-07275-w)
Supplement: Supplementary file 1 — (DOCX 1297 kb) [file 330_2020_7275_MOESM1_ESM.docx]

# Supplementary appendix

### Pre-test metrics

#### Selection

Sociodemographic data were collected by medical interview and self-administered questionnaire, at baseline. In addition, all participants underwent pulmonary function test (PFT), blood sample collection, and smoking cessation program for primary prevention.(1)

Following the selection of subjects by NLST thresholds of age and smoking burden, we selected further robust pre-test metrics for this analysis: gender, smoking status (former/current), and PFT.(2) Other sociodemographic data were excluded because of expected heterogeneous quality (e.g. asbestos exposure).(3) The PFT metrics were selected as follows:(4)

- percent of predicted forced expiratory volume in first second (FEV_1_%_pred_), with threshold 90%;
- the ratio between FEV_1_ and the forced vital capacity (FVC), hereon referred to as FEV1/FVC ratio, with threshold 70%.

The relative risk (RR) in pre-test metrics was calculated by using the category with lower risk of LC cancer. Reference categories are thereafter detailed for each pre-test metric:

- gender: female
- smoking status: former smoker
- FEV_1_%_pred_: > 90%
- FEV_1_/FVC: > 70%

#### Diagnosis of LC

There was association between LC and altered pulmonary function: decreased FEV1%_pred_ and FEV1/FVC was more represented among screenees with LC ([Table 1](#_Table_1)). Increased RR was seen in male gender (3.88-2.72 and smoking status (1.40-1.09). At each time point, the RR for lung cancer was increased in case of altered functional profile (FEV1%_pred_ ≤90% RR 7.444.09; FEV1/FVC ≤ 70% RR 4.49-4.05; [Table 1](#_Table_1_2)).

Pulmonary function tests were the strongest pre-test risk factors for LC, followed by gender. The Kaplan-Meier curves by pre-test metrics are reported in [Supplementary Figure 2A-D](#_Supplementary_figure_2A-D). In particular, FEV1%_pred_ showed HR 7.51 (95%CI 2.60 to 21.75; p = 0.0002) at 1 year, with progressive reduction. Otherwise, FEV1/FVC was steadily associated with HR around 4, independently from the period of observation.

A post-hoc analysis of pre-test metrics in the negative LDCT group did not display intra-group stratification of risk. The Kaplan-Meier curves of pre-test metrics in the negative LDCT group are reported in [Supplementary Figure 3A-D](#_Supplementary_figure_3A-D).

#### Univariate analysis of LC risk

Both pre-test metrics and LDCT outcome were risk factors for LC, by univariate analysis ([Supplementary table 1](#_Table_2A_–)).

Risk of LC was persistently higher in case of abnormal pulmonary function test, notably FEV1%_pred_ ≤ 90% yielded OR 7.62 (95%CI 2.14 to 27.16; p = 0.0017) at in the 1-year analysis. FEV1/FVC ≤ 70% constantly showed OR above 4, as compared with screenees without airflow obstruction. Otherwise, LC risk was marginally influenced by gender and did not vary by smoking status, for any established time point.

#### Multivariate analysis of LC risk

Among pre-test metrics, only FEV1/FVC maintained significant prediction of LC risk in multivariate model, exclusively for the analysis of 3-year interval (Table 2). The analysis was also performed in the selected population of subjects with negative LDCT result, as detailed in Supplementary table 2.

### Simulation of screening algorithms for increased round interval

|  | Negative Baseline LDCT | Indeterminate Baseline LDCT | Positive Baseline LDCT |
| --- | --- | --- | --- |
| Algorithm 1 | LDCT in 2 years | LDCT in 6 months | LDCT in ≤ 3 months |
| Algorithm 2 | LDCT in 2 years | LDCT in 1 year | LDCT in ≤ 3 months |
| Algorithm 3 | LDCT in 3 years | LDCT in 6 months | LDCT in ≤ 3 months |
| Algorithm 4 | LDCT in 3 years | LDCT in 1 year | LDCT in ≤ 3 months |

### Supplementary figure 1

Relative distribution of LDCT outcome and LC diagnosis at 1, 2, and 3 years.


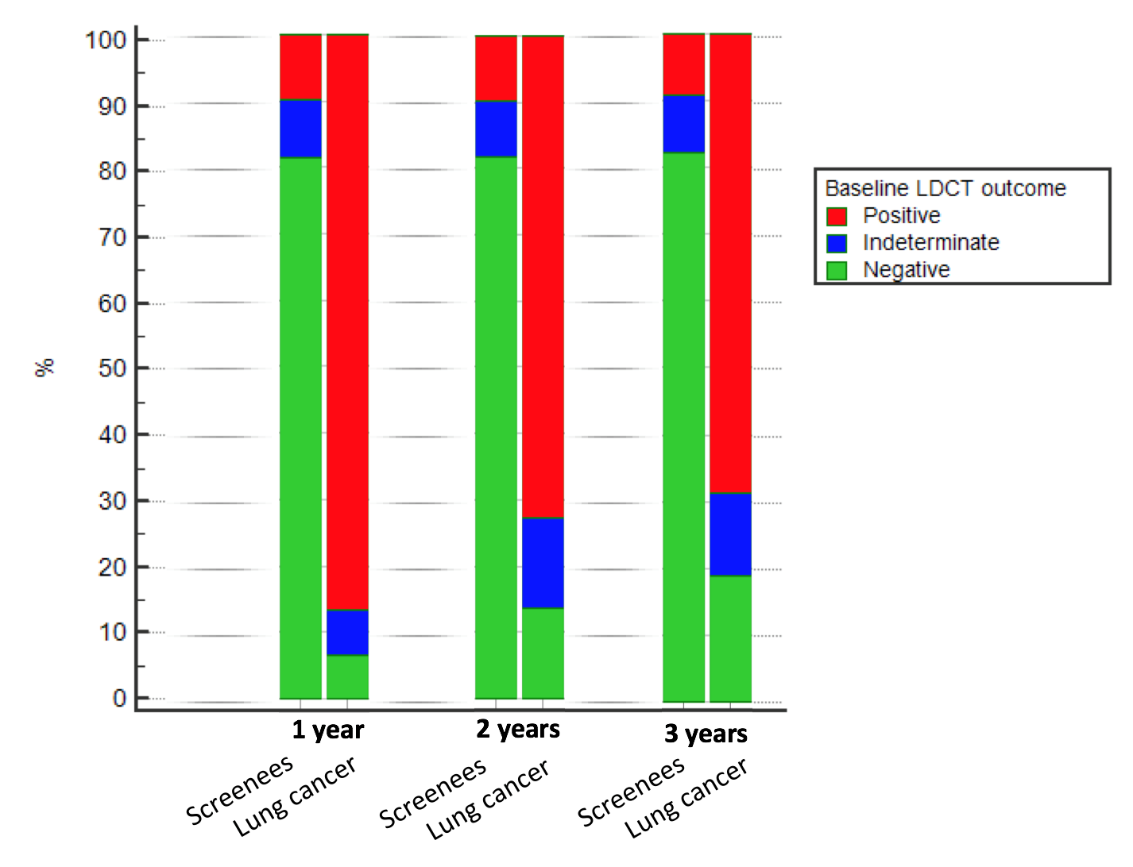


### Supplementary figure 2A-D

Kaplan-Meier curve for diagnosis of lung cancer through the 3 years interval, by pre-test metrics (A: gender; B: smoking status; C: FEV1%_pred_; D: FEV1/FVC). Displayed range of LC probability 0-20%. The bottom section of each figure shows the HR by each predefined time point analysis of LC probability.


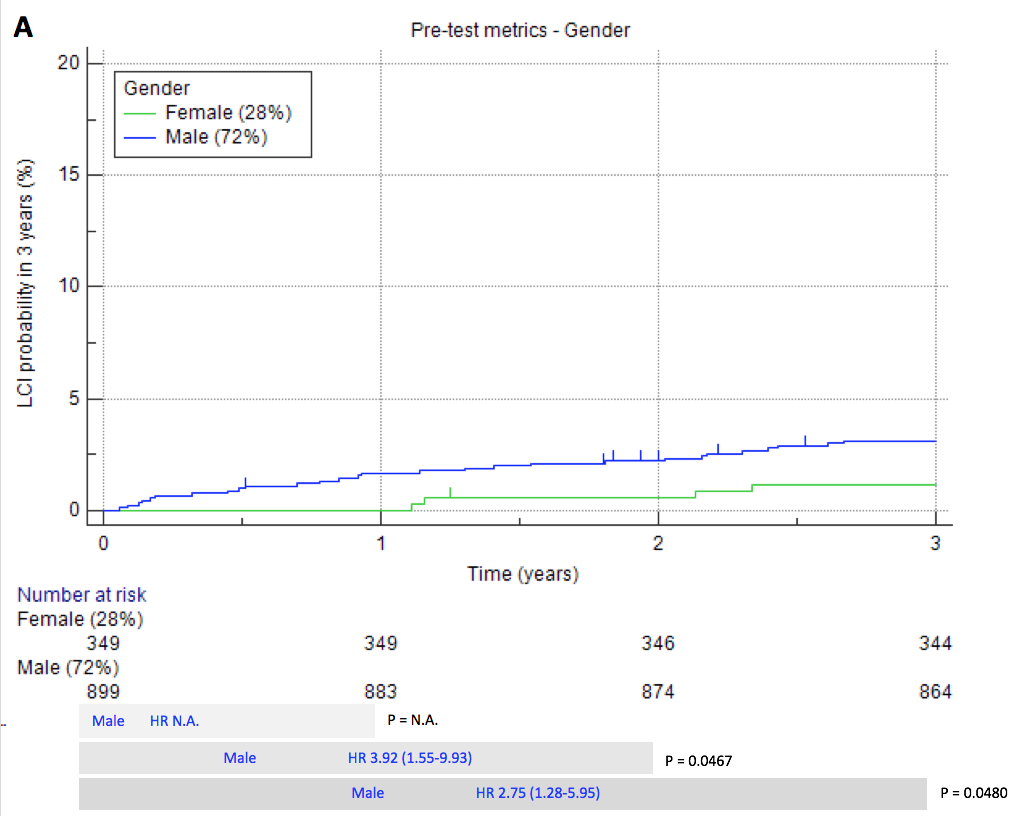


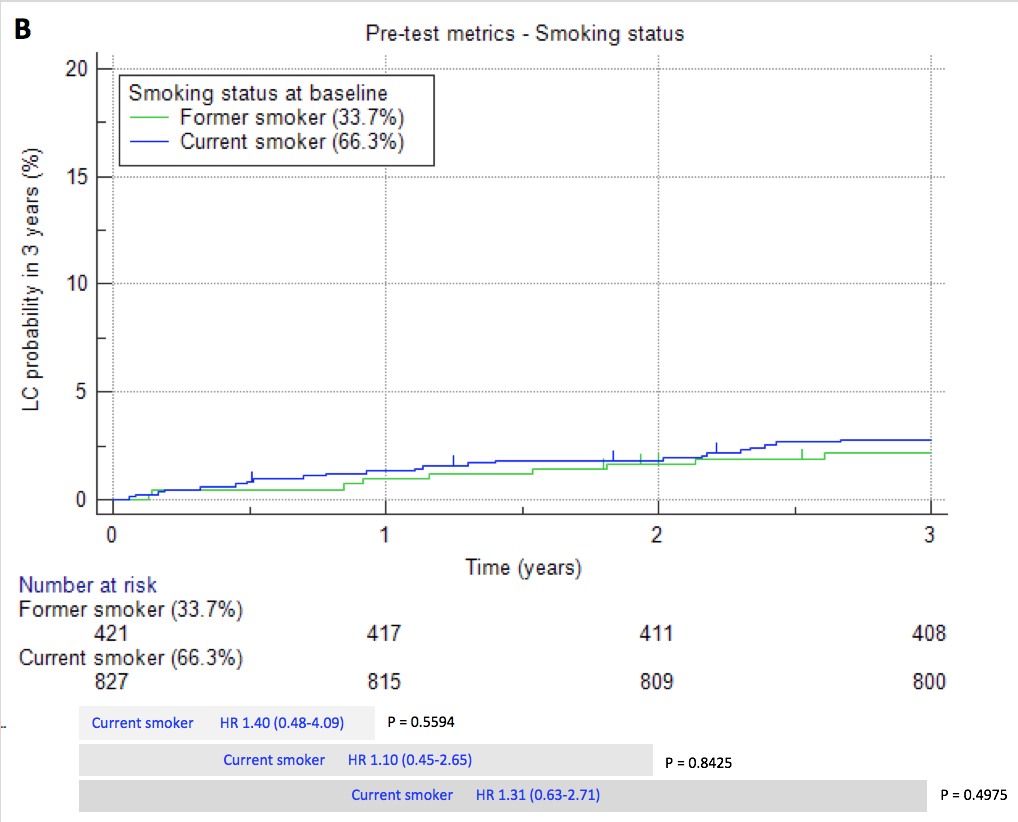


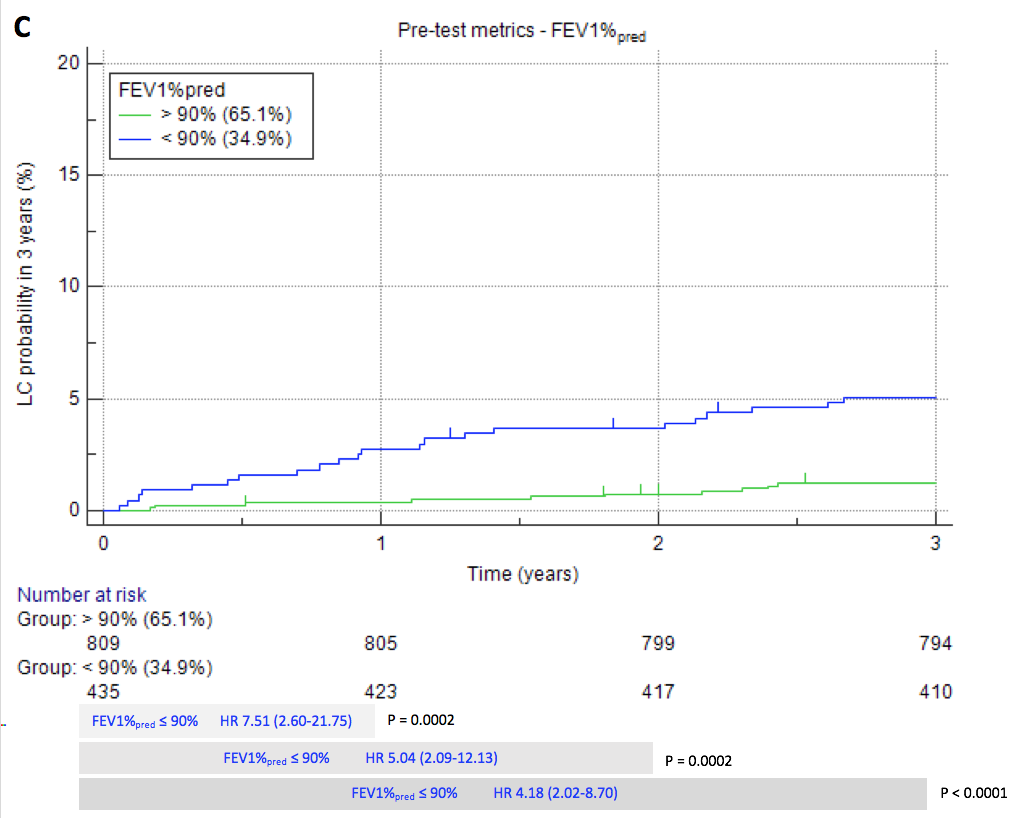


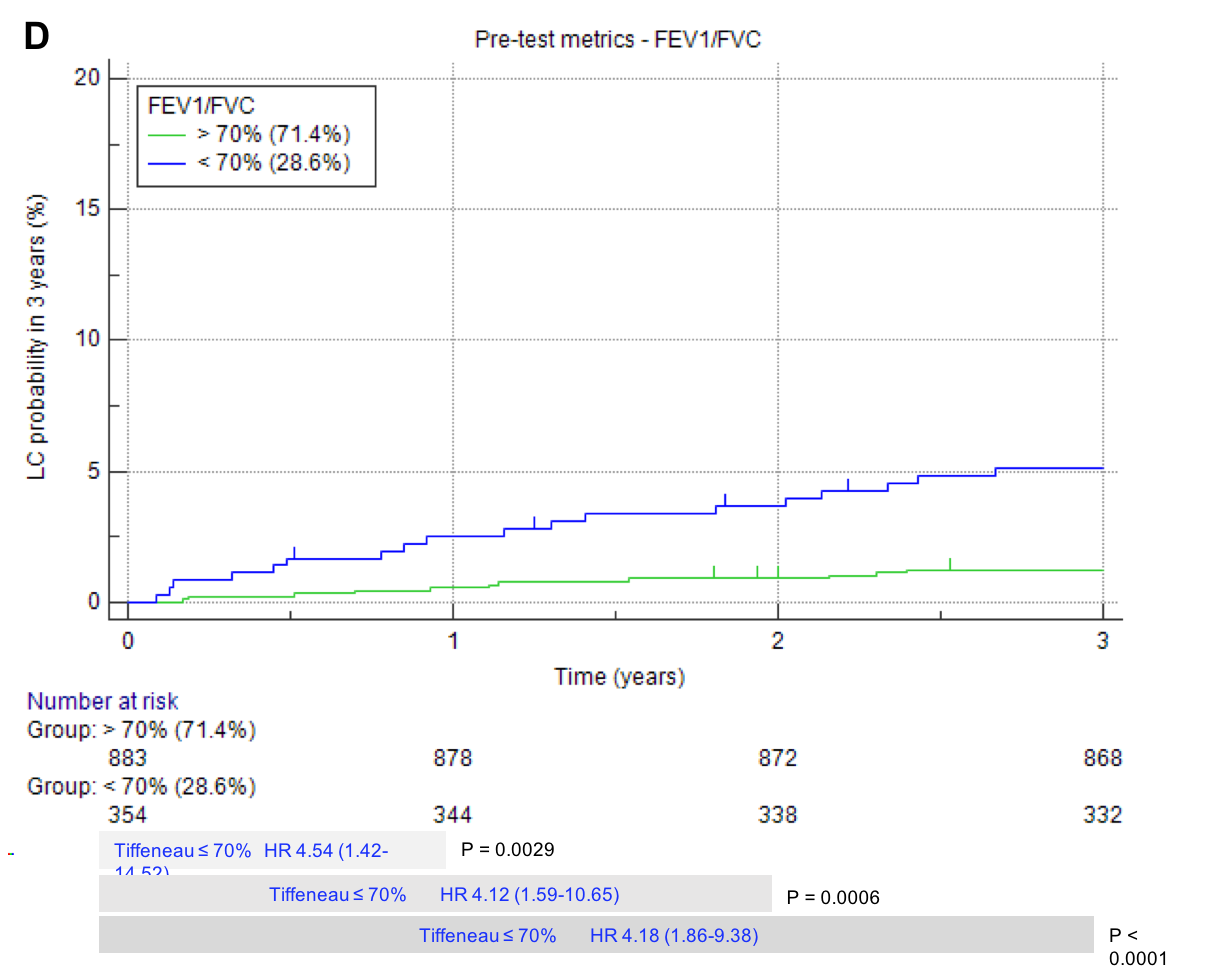


### Supplementary figure 3A-D

Kaplan-Meier curve for diagnosis of lung cancer through the 3 years interval, by pre-test metrics in the subgroup of negative LDCT (A: gender; B: smoking status; C: FEV1%_pred_; D: FEV1/FVC). Displayed range of LC probability 0-2%. The bottom section of each figure shows the HR by each predefined time point analysis of LC probability.


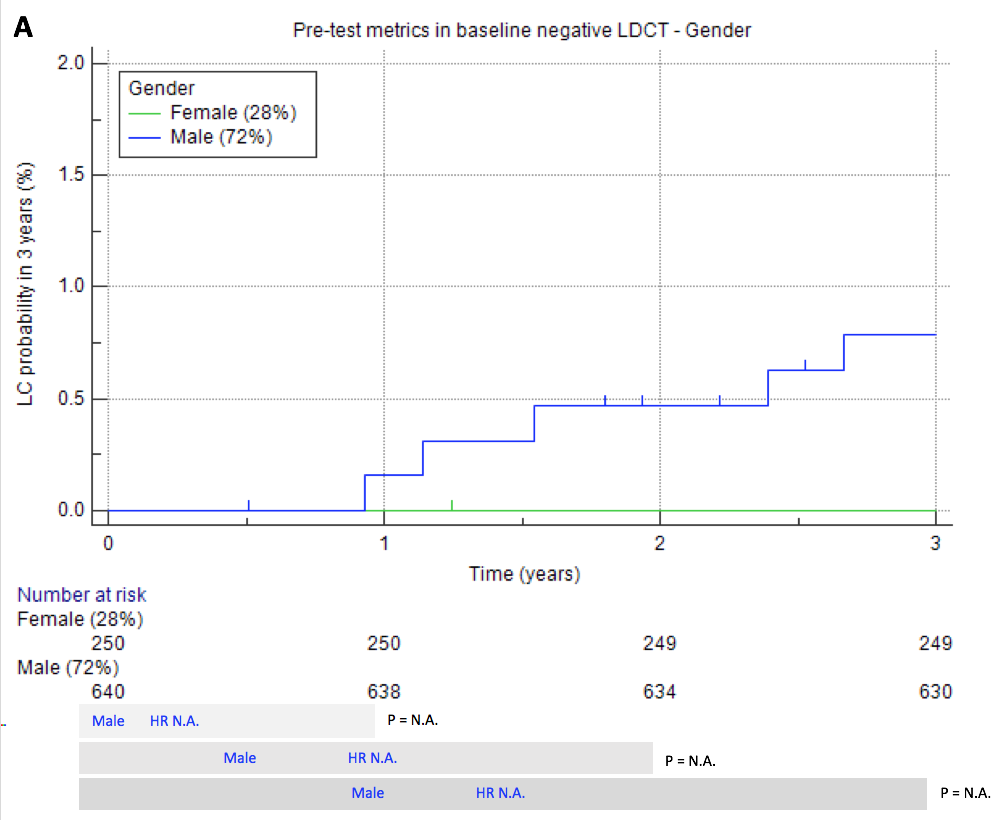


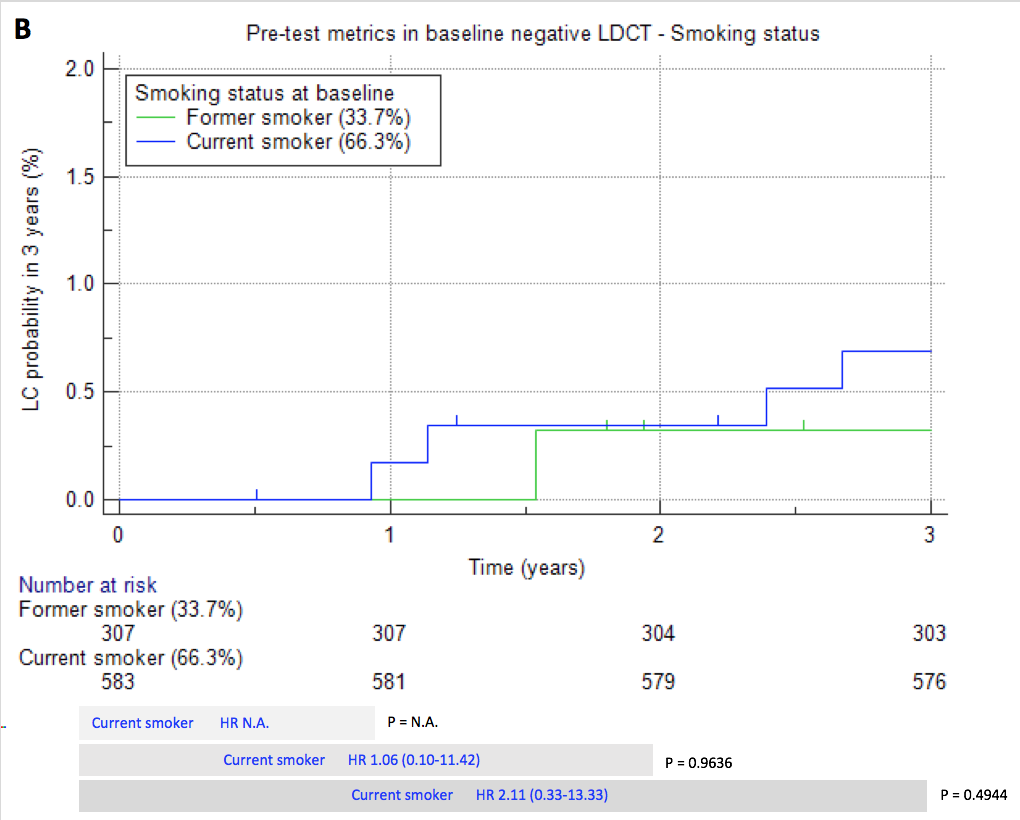


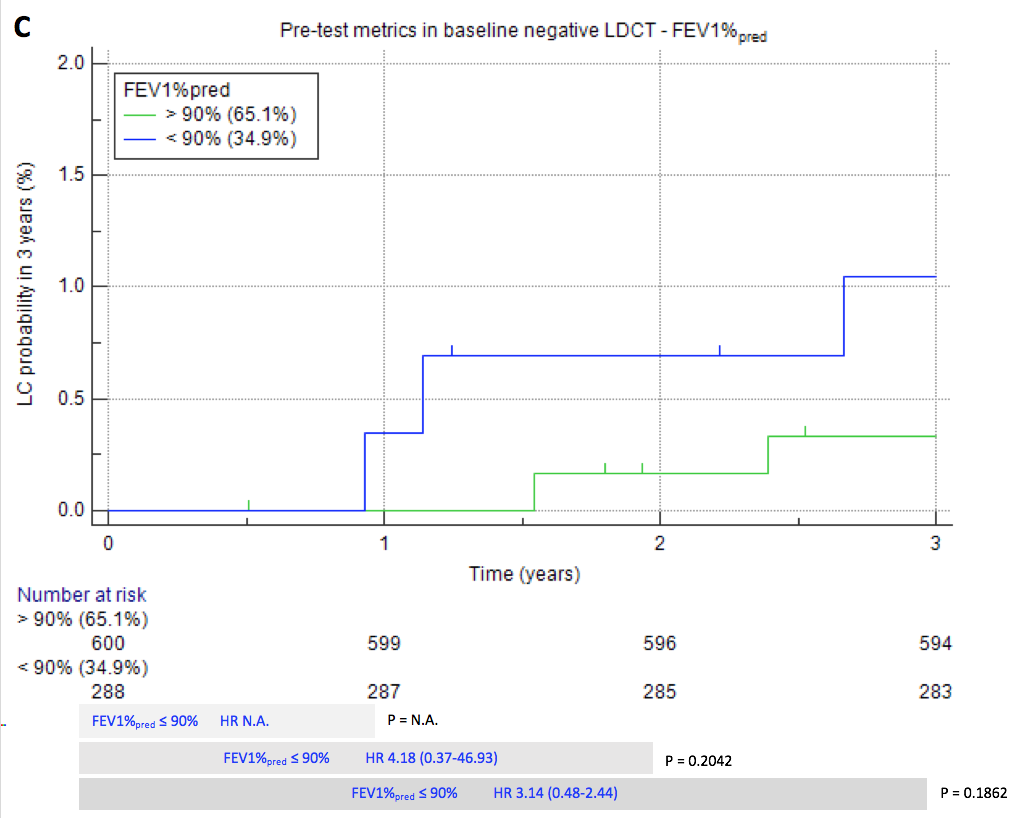


*Number at risk: 2 screenees with negative LDCT outcome and missing FEV1%_pred_ (0 LC through the 3 years).*


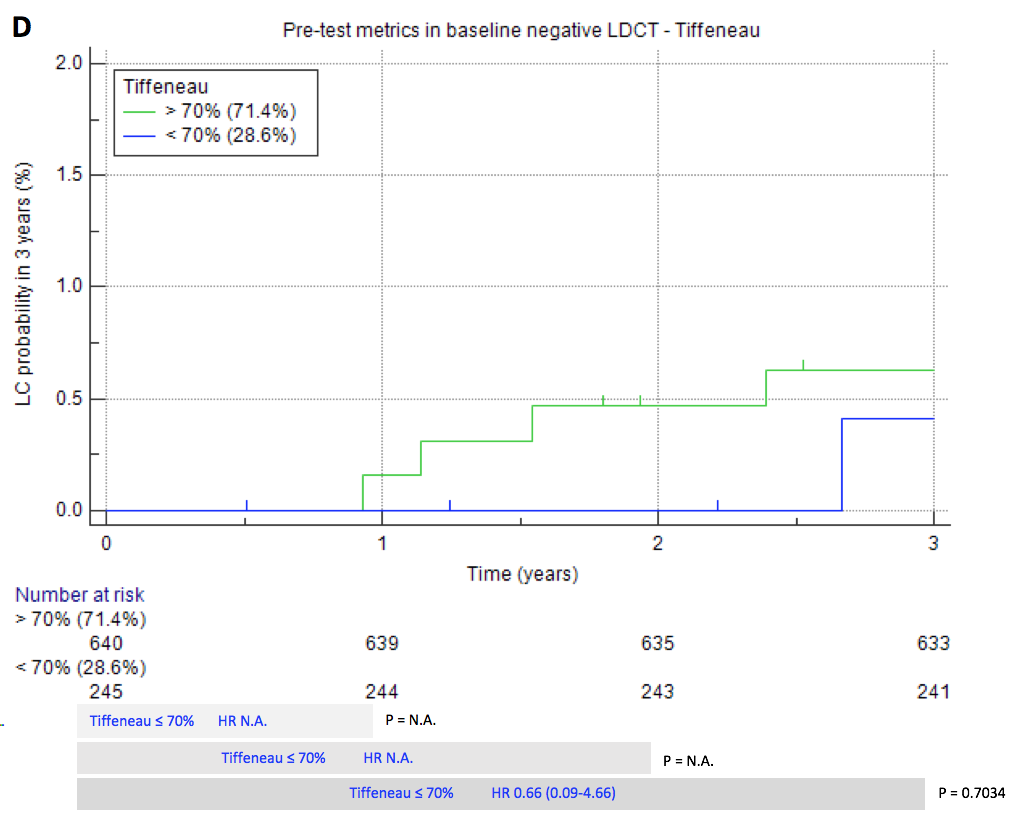


*Number at risk: 5 screenees with negative LDCT outcome and missing FEV1/FVC ratio (0 LC through the 3 years).*

### Supplementary table 1

Univariate analysis of risk of LC diagnosis at 1 year, 2 years and 3 years, according to pre-test metrics and baseline LDCT outcome.

|  | **1 Year** | | **2 Years** | | **3 Years** | |
| --- | --- | --- | --- | --- | --- | --- |
|  | OR (95%CI) | *P* | OR (95%CI) | *P* | OR (95%CI) | *P* |
| **Pre-test metrics** | | | | | | |
| **Gender*** |  |  |  |  |  |  |
| Female | ref |  | ref |  | ref |  |
| Male | N.A. | *-* | 3.92 (0.92-16.67) | *0.0654* | 2.77 (0.97-7.96) | *0.0582* |
| **Smoking Status** |  |  |  |  |  |  |
| Former | ref |  | ref |  | ref |  |
| Current | 1.41 (0.45-4.44) | *0.5624* | 1.10 (0.45-2.67) | *0.8426* | 1.31 (0.6-2.86) | *0.498* |
| **FEV1%_pred_** |  |  |  |  |  |  |
| >90% | ref |  | ref |  | ref |  |
| ≤90% | 7.62 (2.14-27.16) | *0.0017* | 5.04 (1.98-12.81) | *0.0007* | 4.26 (2-9.07) | *0.0002* |
| **FEV1/FVC** |  |  |  |  |  |  |
| >70% | ref |  | ref |  | ref |  |
| ≤70% | 4.58 (1.52-13.77) | *0.0067* | 4.12 (1.72-9.90) | *0.0016* | 4.25 (1.99-9.09) | *0.0002* |
| **Baseline LDCT outcome** | | | | | | |
| Negative | ref |  | ref |  | ref |  |
| Indeterminate | 9.42 (0.60-148.44) | *0.1128* | 9.47 (1.93-46.53) | *0.0059* | 6.36 (1.81-22.39) | *0.0042* |
| Positive | 102.06 (13.49-772.19) | *< 0.0001* | 42.58 (12.48-145.23) | *< 0.0001* | 29.98 (12.21-73.62) | *< 0.0001* |
|  | | | | | | |
| * OR could not be calculated because of absence of LC in one category of the selected metric. | | | | | | |
| OR: odds ratio; 95%CI: 95% confidence interval. | | | | | | |

### Supplementary table 2

Multivariate analysis of risk by pre-test metrics in subjects with negative LDCT result.

|  |  | **1 Year** | | **2 Years** | | **3 Years** | |
| --- | --- | --- | --- | --- | --- | --- | --- |
|  | N  (%) | OR (95%CI) | *P* | OR (95%CI) | *P* | OR (95%CI) | *P* |
| **Pre-test metrics** | | | | | | | |
| **Gender ^a^** | 1,006 |  |  |  |  |  |  |
| Female | 296  (29.4%) | ref |  | ref |  | ref |  |
| Male | 710  (70.6%) | N.A. | *-* | N.A. | *-* | N.A. | *-* |
| **Smoking Status** | 1,006 |  |  |  |  |  |  |
| Former | 342 (33.7%) | ref |  | ref |  | ref |  |
| Current | 664 (66.3%) | N.A. **^a^** | *-* | 1.10  (0.09-12.21) | *0.9382* | 3.01  (0.35-25.77) | *0.3151* |
| **FEV1%_pred_** | 1,004 ^b^ |  |  |  |  |  |  |
| >90% | 675 (67.2%) | ref |  | ref |  | ref |  |
| ≤90% | 329 (32.8%) | N.A. **^a^** | *-* | 6.76  (0.61-74.92) | *0.1216* | 2.47  (0.48-12.79) | *0.2846* |
| **FEV1/FVC** | 1,001 ^c^ |  |  |  |  |  |  |
| >70% | 727 (72.6%) | ref |  | ref |  | ref |  |
| ≤70% | 274 (27.4%) | N.A. **^a^** | *-* | NA. **^a^** | *-* | 0.36  (0.04-3.24) | *0.3630* |
|  | | | | | | | |
| ^a^ OR could not be calculated because of absence of LC in one category of the selected metric. | | | | | | | |
| ^b^ 2 screenees with negative LDCT outcome and missing FEV1%_pred_ (0 LC through the 3 years) | | | | | | | |
| ^c^ 5 screenees with negative LDCT outcome and missing FEV1/FVC ratio (0 LC through the 3 years) | | | | | | | |
| OR: odds ratio; 95%CI: 95% confidence interval. | | | | | | | |

# Supplementary references

1. Pastorino U, Boffi R, Marchiano A, et al. Stopping Smoking Reduces Mortality in Low-Dose Computed Tomography Screening Participants. Journal of thoracic oncology : official publication of the International Association for the Study of Lung Cancer. 2016;11(5):693-9.

2. Sverzellati N, Calabro E, Randi G, et al. Sex differences in emphysema phenotype in smokers without airflow obstruction. The European respiratory journal. 2009;33(6):1320-8.

3. Silva M, Sverzellati N, Colombi D, et al. Pleural plaques in lung cancer screening by low-dose computed tomography: prevalence, association with lung cancer and mortality. BMC Pulm Med. 2017;17(1):155.

4. Muller DC, Johansson M, Brennan P. Lung Cancer Risk Prediction Model Incorporating Lung Function: Development and Validation in the UK Biobank Prospective Cohort Study. Journal of clinical oncology : official journal of the American Society of Clinical Oncology. 2017;35(8):861-9.
